# Does Xpert® MTB/RIF assay give rifampicin resistance results without identified mutation? Review of cases from Addis Ababa, Ethiopia

**DOI:** 10.1186/s12879-020-4817-2

**Published:** 2020-01-30

**Authors:** Ayinalem Alemu, Mengistu Tadesse, Getachew Seid, Helina Mollalign, Kirubel Eshetu, Waganeh Sinshaw, Yeshiwork Abebaw, Misikir Amare, Biniyam Dagne, Getu Diriba, Bazezew Yenew, Melak Getu, Betselot Zerihun

**Affiliations:** Ethiopian Public Health Institute, Addis Ababa, Ethiopia

**Keywords:** Rifampicin-resistance, Molecular Beacon, DNA probes, Xpert® MTB/RIF assay

## Abstract

**Background:**

Xpert® MTB/RIF assay is currently used in Ethiopia for the rapid diagnosis of *Mycobacterium tuberculosis* (*MTB*) and mutations that confer Rifampicin resistance. Rifampicin resistance is determined based on any mutation in the 81 bp of *rpoB* gene using five overlapping probes represented as Probe A (codons 507–511), Probe B (codons 512–518), Probe C (codons 518–523), Probe D (codons 523–529) and Probe E (codons 529–533). In this review, we assessed the frequency of missed probe types for Rifampicin Resistance results.

**Methods:**

Data were reviewed from specimens received and tested using Xpert® MTB/RIF assay at Ethiopian National Tuberculosis Reference Laboratory, in Addis Ababa from 15 July 2016 to 31 December 2018 retrospectively. All archived data were reviewed carefully to describe missed probe types and the quantity of DNA in the sample.

**Results:**

A total of 100 specimens were reported as *MTB Detected Rifampicin Resistance Detected* by Xpert® MTB/RIF assay. More than half (55%) of these results were reported from male patients. The median age was 28.0 years (5 months to 88 years). Majorities (62%) of the cases were detected from sputum. Among the total of 38 extrapulmonary samples, lymph node aspirates were accounted for 50% (19/38). The most common mutations (81.0%) were found in the Probe E region followed by Probe D (10.0%), and Probe B (3.0%). Mutations in Probe A and Probe C regions were not observed. However, six (6.0%) Rifampicin resistance cases were found without any missed probe type. The delta Ct max is ≥4.3. No specimen yielded Rifampicin resistance associated with more than one probe failure or mutation combinations.

**Conclusion:**

Mutations associated with Probe E (codons 529–533) region were identified as the commonest *rpoB* gene mutations. The Rifampicin resistance results found without any identified missing probe needs further study. The lower DNA amount was observed in extrapulmonary specimens compared with sputum.

## Background

World health organization (WHO) endorsed Xpert® MTB/RIF assay for the diagnosis of *Mycobacterium tuberculosis* (*MTB)* and the mutations that confer Rifampicin resistance (RR) [1]. This assay has revolutionized the diagnosis of TB by simultaneously detecting the bacteria and RR [2, 3], which is a surrogate marker for MDR-TB [2, 4]. RR is determined based on mutations in the 81 bp (codons 507–533) regions of the *β*-subunit of the RNA polymerase enzyme *(rpoB)* gene using five overlapping probes [3, 5]. These probes are named as Probe A (codons 507–511), Probe B (codons 511–518), Probe C (codons 518–523), Probe D (codons 523–529) and Probe E (codons 529–533) [3, 5]. A mutation in these regions accounts for more than 95% of RR [3, 4].

Ethiopia is among the 30 high TB, TB/HIV and MDR-TB burdened countries in the world [1]. In 2017, there was an estimated TB incidence of 164/100, 000 population. In the same year, MDR/RR-TB rate was estimated to be 2.7% among new cases and 14% among previously treated cases [1]. Ethiopia has been implementing Xpert® MTB/RIF assay for the diagnosis of TB and RR-TB which provided encouraging results [6]. Studies showed that the probe that confers RR is rarely reported in the clinical practice of many countries [5]. Similarly, there is no practice in reporting RR-TB with the type of missed probe and the Ct values in Ethiopia, specifically the current study setting. Identification of the nature of *rpoB* mutation could provide useful information for accurate diagnosis of RR-TB and when there is a need to studying the epidemiology of RR-TB in a particular region [3, 5]. However, information on the frequency of *rpoB* gene mutations in this study setting is scarce. Therefore, this review aimed to provide relevant information on the frequency of associated mutations for RR results using Xpert® MTB/RIF assay in Addis Ababa, Ethiopia.

## Methods

### Setting

Data were reviewed retrospectively from archived result log sheet for specimens received and tested using Xpert® MTB/RIF assay at Ethiopian National Tuberculosis Reference Laboratory (NTRL) from 15 July 2016 to 31 December 2018**.** NTRL is an accredited national reference laboratory located in Addis Ababa, Ethiopia, which provides different services including diagnostic testing using Xpert® MTB/RIF assay. Retrospective data were reviewed from archived logbooks and databases. The assay performed using Xpert® MTB/RIF assay (Cepheid, Sunnyvale, CA, USA). Socio-demographic and clinical data were reviewed for patients with RR-TB results. Important details were accessed from the database.

### Laboratory diagnosis

Sputum samples and extrapulmonary specimens [lymph node aspirates, pleural fluid, pus, abscess, ascetic fluid and bronco alveolar leverages (BAL)] were collected from TB presumptive patients. Samples were transported by triple packaging system through trained couriers within acceptable (2-8^o^c) temperature conditions to NTRL. Approximate of 4 ml sputum was mixed with 8 ml of sample reagent buffer (supplied within the kit), shaken the tubes vigorously 20 times and allowed to stay for 10 min. Following this, it was mixed again and stayed for 5 min. Later, an approximate of > 2 ml (not more than 4 ml) of the specimen was dispensed into Xpert MTB/RIF’s cartridge and loaded into the GeneXpert instrument (Cepheid, Sunnyvale, CA, USA). The result was released after 2 h. For extrapulmonary samples, different approaches were used based on the nature of the specimen. For example, lymph node samples were decontaminated by using a 3% NALC-NaOH method and the sediment was used for the test in 1:3 ratios (0.5 ml sediment and 1.5 ml sample reagent buffer) as done elsewhere [7].

Results fromXpert® MTB/RIF assay were categorized into three result types such that; *Mycobacterium tuberculosis* not detected*, Mycobacterium tuberculosis* detected and Error/Invalid/No results. Along with all *Mycobacterium tuberculosis* detected test results Rifampicin resistance was determined. For all test results having *Mycobacterium tuberculosis* detected results, probe types and DNA amounts were assessed. Such that all RR-TB test results were archived from the GeneXpert instrument and exported to a database. All the information including the missed probe types and level of each DNA amount were reviewed from the database and crosschecked on the GeneXpert instrument because the GeneXpert instrument can give all details of test results in PDF format.

### Statistical analysis

Extracted data were checked for completeness, coded, entered and analyzed by using Statistical Packages for Social Sciences (SPSS) Version 20. Descriptive statistics were used to characterize demographic and clinical variables. Frequency of specimen types, test results, DNA amounts, and missed probe types were determined.

## Results

### Demographic and clinical information

**Fig. 1:**
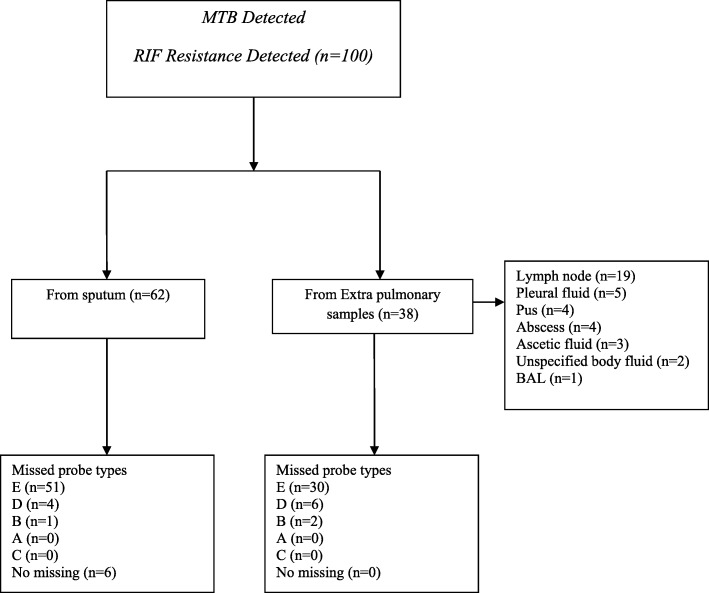
Flowchart of Rifampicin resistant results using the Xpert® MTB/RIF assay

Among all specimens processed by Xpert® MTB/RIF assay at NTRL during the review period, *MTB detected Rifampicin resistance detected* was reported from 100 specimens. Demographic characteristics and treatment history were collected for TB patients having RR results and more than half (55%) were males. The majority of the patients were found in the age group of 25–34 years. Among all 100 RR-TB patients, half (50) were new patients and 29 were relapses, while the rest 11 were either failures or return after loss to follow up. The majority (62, 62%) of the specimens were sputum, while the remaining 38(38%) were extrapulmonary samples. Among the total of 38 extrapulmonary samples, lymph node aspirates were accounted 50% (19, 50%) followed by pleural fluid (5, 13.2%), pus (4, 10.5%), abscess (4, 10.5%), ascetic fluid (3, 8.0%), unspecified body fluids (2, 5.2%) and BAL (2, 2.6%) (Table 1) (Fig. 1).

**Table 1:** Demographic and clinical characteristics of study participants (*n* = 100)

Characteristics	Number	Proportion
Age group		
< 14	5	5%
15–24	29	29%
25–34	35	35%
35–44	17	17%
45–54	6	6%
55–64	4	4%
> 64	2	2%
Missed	2	2%
Total	100	100%
Sex		
Male	55	55%
Female	45	45%
Total	100	100%
Treatment history		
New	50	55.6%
Relapse	29	32.2%
Failure	7	7.8%
A loss to follow up	4	4.4%
Total	90	100.0%
Specimen type		
Sputum	62	62.0%
Extrapulmonary	38	38.0%
Total	100	100.0%
Extrapulmonary samples		
Lymph node	19	50.0%
Pleural fluid	5	13.2%
Pus	4	10.5%
Abscess	4	10.5%
Ascetic fluid	3	8.0%
Unspecified body fluid	2	5.2%
BAL	1	2.6%
Total	38	100.0%

### Missed probe types

As Xpert® MTB/RIF assay gives the amount of DNA semi-quantitatively, the DNA amount of RR results was found as a very low (14, 14%), low (23, 23%), medium (36, 36%) and high (27, 27%). Of the 63 medium or high DNA amount test results, majority 51 (80.95%) were from sputum samples, whereas among the 37 low or very low DNA amount results, majority 24 (64.87%) were from EPTB samples (Table 2). The commonest mutation was located in probe E (codons 529–533) region (81, 81%) followed by probe D region (codons 523–529) (10, 10%) and probe B region (codons 512–518) (3, 3%). There was no mutation associated with Probe A (codons 507–511) and Probe C (codons 518–523) regions (Fig. 1). The delta Ct max was ≥4.3. However, six (6, 6.0%) RR-TB cases were found without any identified missed probe type. All these six RR results were detected from sputum samples and of which five were from new patients (Table 2).

**Table 2:** Distribution of missed probe types across clinical factors of RR-TB patients (*n* = 100)

Characteristics	Missed probes types, N				Number	Proportion
	B	D	E	No missed probe		
Treatment history						
New	1	7	37	5	50	55.6%
Relapse	2	25	1	1	29	32.2%
Failure	–	–	7	–	7	7.8%
A loss to follow up	–	1	3	–	4	4.4%
Total	3	33	48	6	90	100.0%
Specimen type						
Sputum	1	4	51	6	62	62.0%
Extrapulmonary	2	6	30	–	38	38.0%)
Total	3	10	81	6	100	100.0%
Extrapulmonary samples						
Lymph node	2	2	15	–	19	50.0%
Pleural fluid	–	2	3	–	5	13.2%
Pus	–	–	4	–	4	10.5%
Abscess	–	–	4	–	4	10.5%
Ascetic fluid	–	–	3	–	3	8.0%
Unspecified body fluid	–	1	1	–	2	5.2%
BAL	–	1	–	–	1	2.6%
Total	2	6	30	–	38	100.0%
GeneXpert semi-quantification of *MTB*						
Very low	–	–	11	3	14	14%
Low	1	3	19	–	23	23%
Medium	1	5	29	1	36	36%
High	1	2	22	2	27	27%
Total	3	10	81	6	100	100%

“-” = Not available, N=Number

## Discussion

In addition to the simultaneous detection of *MTB* and its resistance to Rifampicin, Xpert® MTB/RIF assay might be used to understand the molecular epidemiology of *MTB* and to identify hot spots of drug-resistant TB transmission. Demographic characteristics and treatment history were collected for all 100 RR-TB patients and more than half of the RR-TB patients were males, where it was reported previously that males are highly affected by TB compared to females [1, 8]. In a systematic review conducted in Ethiopia, it was also reported that being male has been identified as a risk factor for multi-drug resistant tuberculosis [9]. The most highly affected age groups were productive individuals found in the age group of 25–34 years, which is comparable with various studies [1, 10–12]. This might be due to exposure to open cases of TB where young individuals especially males are prone to TB associated risk factors.

Although the Xpert® MTB/RIF assay was optimized for the respiratory specimen [13], this study showed that the assay can provide valid results in extrapulmonary samples. Among 100 RR-TB results, the majority (62) was detected from sputum samples, while the remaining 38 samples were detected from different types of extrapulmonary samples. From the extrapulmonary samples, half was a lymph node aspirate sample which is similar to the study reported from Dessie, Ethiopia [14]. In this review higher DNA amount (high or medium) was observed in the sputum samples, while lower DNA amount (low or very low) was observed in extrapulmonary samples. This lower DNA amount in EPTB samples might cause false-negative results [15–17] which should be considered while preparing specimens for Xpert® MTB/RIF assay to increase sensitivity.

Rifampicin resistance is determined in Xpert® MTB/RIF assay by *rpoB* gene mutations in the 81 bp-RRDR of *MTB* which are five overlapping regions labeled as A (codons 507–511), B (codons 511–518), C (codons 518–523), D (codons 523–529) and E (codons 529–533) [3–5]. In this review, the commonest mutation was located in codons covering 529–533 which is represented by Probe E (81, 81%). This was also reported by previous studies conducted in Ethiopia [18–20]. Similarly, in the previous studies done in Africa countries in Nigeria [21] and in Uganda [2], mutations conferring RR are located in mostly the region of Probe E. Likewise, in studies done at Asian countries in India [3, 5], Pakistan [4] and Bangladesh [22] missing of probe E was predominant. However, Most of the RR cases detected by Xpert® MTB/RIF assay were associated with probe B (23/64) and probe E (23/64) in a study done in Malawi [23]. The information about the probes conferring RR could be used to assess trends over time, identify pockets of transmission, or investigate outbreaks, especially when the RR is secondary to mutations outside the Probe E region. In this review following probe E the proportion of each missed probe were: probe D (10%) and probe B (3%). This was also observed in a study done in Nigeria [21]. However, most of the previous studies conducted in African and Asian regions indicate that following Probe E the most common mutations conferring RR are located in the region was Probe B followed by Probe D [2–5, 22]. In a study done in Malawi, the proportion of mutation in Probe E and Probe B is equal [23]. In this review there was no mutation associated with Probe A and probe C, probably this particular site of RRDR is less susceptible to mutations conferring this resistance or might be the less common mutation of these probes in this particular area (Addis Ababa). Similarly, the absence of Probe C mutation was reported from Nigeria [21] and from Uganda [2]. Likewise, it was also reported that mutations in Probe A and Probe C were less common in other studies [3–5, 22, 23].

In this review, we found that six (6, 6%) test results were resistant to Rifampicin without any identified missed probe. The possible reason behind this could be the delta Ct (ΔC_T_) max. Delta Ct max is the difference between the first (early C_T_) and the last (late C_T_) *MTB* specific beacon [16]. In the Xpert® MTB/RIF assay, for *MTB Detected Rifampicin Resistance Detected/RR-TB/* test results the delta Ct max should be > 4 [24]. This has happened in the current review where the ΔC_T_ max was ≥4.3. However, it needs further study or clarification. In addition, it was reported previously that, the amount of DNA affects Rifampicin resistance results in the Xpert® MTB/RIF assay [25]. Even though not used in this review and previous studies, the codon used to detect Rifampicin resistance could be used for contact tracing. Berhanu et al reported that a Rifampicin resistant discordant result in Xpert® MTB/RIF assay was associated with Probe B [26].

The limitation of this review was that no gold standard (phenotypic DST and sequencing) method was used for the comparison of Xpert® MTB/RIF assay results to estimate the proportion of false drug resistance or susceptibility. Furthermore, as a retrospective review, it lacks other relevant variables such as contact history, *HIV* status, vaccination status and location of the household district.

## Conclusion

Mutations associated with Probe E (codons 529–533) are identified as the commonest *rpoB* gene mutation in Ethiopia and other countries as identified in this and previous studies. In the reviewed data, mutations associated with Probe A (codons 507–511) and Probe C (codons 518–523) are not identified. RR-TB was found without any missing probes in six sputum samples (6%) which necessitate further study or investigation. The lower DNA amount is observed in extrapulmonary samples compared with sputum samples. A further larger study is needed to confirm RR-TB cases by using gold standard methods (Mycobacterium culture and phenotypic DST).

## Data Availability

All original raw data is available in the corresponding author.

## Abbreviations

DNA: Deoxyribose Nucleic Acid
EPTB: Extra Pulmonary Tuberculosis
*HIV*: *Human Immunodeficiency Virus*
MDR-TB: Multidrug-Resistant Tuberculosis
*MTB*: *Mycobacterium tTuberculosis*
NTRL: National Tuberculosis Reference Laboratory
RNA: Ribose Nucleic Acid
*rpoB*: RNA polymerase enzyme *β*-subunit
RR: Rifampicin Resistance
RRDR: Rifampicin Resistance Determining Region
SPSS: Statistical Package for Social Sciences
TB: Tuberculosis
WHO: World Health Organization

## References

[1] WHO (2018). Global Tuberculosis Report.

[2] Mboowa G, Namaganda C, Sengooba W (2014). Rifampicin resistance mutations in the 81 bp RRDR of *rpoB* gene in Mycobacterium tuberculosis clinical isolates using Xpert® MTB/RIF in Kampala, Uganda: a retrospective study. BMC Infect Dis.

[3] Kaur R, Jindal N, Arora S, Kataria S (2016). Epidemiology of rifampicin-resistant tuberculosis and common mutations in *rpoB* gene of *Mycobacterium tuberculosis*: a retrospective study from six districts of Punjab (India) using Xpert MTB/RIF assay. J Lab Physicians.

[4] Ullah I, Shah AA, Basit A, Ali M, Khan A, Ullah U (2016). Rifampicin resistance mutations in the81 bp RRDR of *rpoB* gene in *Mycobacterium tuberculosis* clinical isolates using Xpert MTB/RIF in Khyber Pakhtunkhwa, Pakistan: a retrospective study. BMC Infect Dis.

[5] Reddy Raghuprakash, Alvarez-Uria Gerardo (2017). Molecular Epidemiology of Rifampicin Resistance in Mycobacterium tuberculosis Using the GeneXpert MTB/RIF Assay from a Rural Setting in India. Journal of Pathogens.

[6] Federal Democratic Republic of Ethiopia Ministry of Health/Ethiopian Public Health Institute (2014). Implementation Guideline for GeneXpert MTB/RIF Assay in Ethiopia.

[7] Tadesse Mulualem, Abebe Gemeda, Abdissa Ketema, Aragaw Dossegnaw, Abdella Kedir, Bekele Alemayehu, Bezabih Mesele, Apers Ludwig, de Jong Bouke C., Rigouts Leen (2015). GeneXpert MTB/RIF Assay for the Diagnosis of Tuberculous Lymphadenitis on Concentrated Fine Needle Aspirates in High Tuberculosis Burden Settings. PLOS ONE.

[8] Horton Katherine C., MacPherson Peter, Houben Rein M. G. J., White Richard G., Corbett Elizabeth L. (2016). Sex Differences in Tuberculosis Burden and Notifications in Low- and Middle-Income Countries: A Systematic Review and Meta-analysis. PLOS Medicine.

[9] Asgedom Solomon Weldegebreal, Teweldemedhin Mebrahtu, Gebreyesus Hailay (2018). Prevalence of Multidrug-Resistant Tuberculosis and Associated Factors in Ethiopia: A Systematic Review. Journal of Pathogens.

[10] Derbie A, Worku S, Mekonnen D, Mezgebu Y, Teshager A, Birhan A, et al. Xpert MTB/RIF assay for the diagnosis of *Mycobacterium tuberculosis* and its Rifampicin resistance at Felege Hiwot and Debre Tabor Hospitals, Northwest Ethiopia: A preliminary implementation research. Ethiop. J. Health Dev. 2016;30(2).

[11] Ejeta E, Beyene G, Bonsab Z, Abebe G (2018). Xpert MTB/RIF assay for the diagnosis of Mycobacterium tuberculosis and rifampicin resistance in high human immunodeficiency virus setting in Gambella regional state, Southwest Ethiopia. J ClinTuberc Other Mycobact Dis.

[12] Mulu W, Abera B, Yimer M, Hailu T, Ayele H, Abate D (2017). Rifampicin-resistance pattern of Mycobacterium tuberculosis and associated factors among presumptive tuberculosis patients referred to Debre Markos referral hospital, Ethiopia: a cross-sectional study. BMC Res Notes.

[13] WHO: Xpert MTB/RIF assay for the diagnosis of pulmonary and extrapulmonary TB in adults and children. Geneva: World Health Organization. 2013; ISBN: 978 92 4 150633 5.

[14] Metaferia Yeshi, Seid Abdurahaman, Fenta Genet Mola, Gebretsadik Daniel (2018). Assessment of Extrapulmonary Tuberculosis Using Gene Xpert MTB/RIF Assay and Fluorescent Microscopy and Its Risk Factors at Dessie Referral Hospital, Northeast Ethiopia. BioMed Research International.

[15] Purohit M, Mustafa T. Laboratory Diagnosis of Extra-pulmonary Tuberculosis (EPTB) in Resource-constrained Setting: State of the Art, Challenges and the Need. J Clin Diagn Res. 2015;9(4). 10.7860/JCDR/2015/12422.5792 PMC4437077 26023563

[16] Lawn SD, Zumla AI (2012). Diagnosis of extrapulmonary tuberculosis using the Xpert® MTB/RIF assay. Expert Rev Anti-Infect Ther.

[17] Kohli M, Schiller I, Dendukuri N, Dheda K, Denkinger CM, Schumacher SG, Steingart KR. Xpert® MTB/RIF assay for extrapulmonary tuberculosis and rifampicin resistance. Cochrane Database Syst Rev. 2018;8. 10.1002/14651858.CD012768.pub2 PMC6513199 30148542

[18] Tessema B, Beer J, Emmrich F (2012). Analysis of gene mutations associated with isoniazid, rifampicin and ethambutol resistance among Mycobacterium tuberculosis isolates from Ethiopia. BMC Infect Dis.

[19] Biadglegne F, Tessema B, Rodloff AC (2013). Magnitude of gene mutations conferring drug resistance in Mycobacterium tuberculosis isolates from lymph node aspirates in Ethiopia. Int J Med Sci.

[20] Tadesse M, Aragaw D, Dimah B, Efa F, Abdella K, Kebede W (2016). Drug resistance-conferring mutations in *Mycobacterium tuberculosis* from pulmonary tuberculosis patients in Southwest Ethiopia. Int J Mycobacteriology.

[21] Ochang EA, Udoh UA, Emanghe UE, Tiku GO, Offor JB, Odo M (2016). Evaluation of rifampicin resistance and 81-bp rifampicin-resistant determinant region of rpoB gene mutations of Mycobacterium tuberculosis detected with Xpert MTB/RIF in Cross River state, Nigeria. Int J Mycobacteriology.

[22] Rahman A, Sahrin M, Afrin S, Earley K, Ahmed S, Rahman SMM (2016). Comparison of Xpert MTB/RIF assay and GenoType MTBDRplus DNA probes for detection of mutations associated with rifampicin resistance in Mycobacterium tuberculosis. PLoS One.

[23] Chikaonda T, Ketseoglou I, Nguluwe N, Krysiak R, Thengolose I, Nyakwawa F, et al. Molecular characterization of rifampicin-resistant *Mycobacterium tuberculosis* strains from Malawi. Afr J Lab Med. 2017;6(2). 10.4102/ajlm.v6i2.463 PMC5523914 28879159

[24] Prakash AK, Datta B, Tripathy JP, Kumar N, Chatterjee P, Jaiswal A. The clinical utility of cycle of threshold value of GeneXpert MTB/RIF (CBNAAT) and its diagnostic accuracy in pulmonary and extra-pulmonary samples at a tertiary care center in India. Int J Tuberc. 2018;283. 10.1016/j.ijtb.2018.05.021 30522616

[25] Ocheretina O, Byrt E, Mabou MM, Mardi GR, Merveille YM, Rouzierb V (2016). bFalse-positive rifampin resistant results with Xpert MTB/RIF version 4 assay in clinical samples with a low bacterial load. Diagn Microbiol Infect Dis.

[26] Berhanu H, Schnippel K, Kularatne R, Firnhaber C, Jacobson KR, Horsburgh CR (2019). Discordant rifampicin susceptibility results are associated with Xpert MTB/RIF probe B and probe binding delay. Int J Tuberc Lung Dis.

